# Improving analysis and use of routine reproductive, maternal, newborn, and child health facility data in low-and middle-income countries: a universal priority

**DOI:** 10.1186/s12913-021-06649-0

**Published:** 2021-09-13

**Authors:** Theresa Diaz, Jennifer Requejo

**Affiliations:** 1 World Health Organization, Department of Maternal, Newborn, Child and Adolescent Health and Ageing, Geneva, Switzerland; 2 United Nations Children’s Fund, Division of Data, Analytics, Planning & Monitoring, New York, NY USA

In February 2020 a meeting of reproductive, maternal, newborn, and child health (RMNCH) measurement experts was convened in Nairobi, Kenya, to reflect on a 2015 call to action for a robust RMNCH measurement system that included five principles: (1) a focus on a core set of global indicators on effective coverage; (2) ensuring selected indicators and data collected on RMNCH are relevant to countries; (3) investment in measurement innovations in methods and instruments to help fill data gaps; (4) undertake equity analyses on intervention coverage and health outcomes to make sure no one gets left behind; and (5) global leadership to drive the development of measurement improvements, strong country health information systems, and increased data use at all levels (global, regional, country) [1, 2]. At the 2020 meeting, it was recognized that the principle of country relevance warranted greater emphasis so a sixth principle was added to the list on improving the use of data to support local action and country ownership of the measurement and accountability agenda. This sixth principle aligns with WHO’s agenda to improve country routine health information systems (RHIS), encompassing public and private health facility data, and increase country uptake of RHIS data for evidence-based decision making around resource allocation and programming [3]. Shortly after this meeting, where participants renewed a pledge to invest in RHIS for RMNCH, COVID-19 was declared a global pandemic and the world drastically changed. Health care systems continue to experience considerable strain in many countries due to the health crisis, mitigation measures put into place to prevent transmission have frequently disrupted access to health services, and fear of possibly contracting SARS-COV2 when visiting health facilities has resulted in lower use of RMNCH services. Although the impact of the pandemic on maternal, newborn, and child mortality is not yet known, modeling efforts have shown how drops in essential health service coverage may result in increased preventable deaths in women and children [4, 5].

Comparable information across countries on health service disruptions and their health impacts has been difficult to collect during the pandemic because of suspensions in population-based household surveys and health facility assessments. To get a general sense of the severity of disruptions across RMNCH services in low-and middle-income countries (LMICs), United Nations agencies and other groups conducted rounds of key informant surveys in 2020 and the first quarter of 2021 [6–8]. These pulse surveys, while informative, suffered from the limitation of being based on subjective reporting, and provided insufficiently detailed assessments of the situation in facilities and communities to guide country programmatic decisions. Global guidance was also rapidly developed by United Nations agencies and partners to support country use of RHIS for monitoring RMNCH service disruptions [9]. Implementation of these guidelines in LMICs with WHO and UNICEF support, however, has proved challenging because of limited data availability in many country RHIS systems, frequent problems with poor data quality, and the lack of capacity of local staff to undertake data quality assessments and make corrections as required. These experiences, coupled with the need for real-time data to develop effective response strategies to the COVID19 crisis, have made irrefutable the importance of strengthening RHIS for rapid and accurate assessments of supply and demand side challenges to health service use. If the global community had provided greater financial resources and technical support to countries to develop their RHIS prior to the pandemic, countries could have used data generated from these systems to develop nimble approaches to addressing the COVID19 crisis that kept essential RMNCH services intact. Governments are also accountable for inadequate investments in health information systems in the years preceding the pandemic, including in human resource capacity to analyze and use RHIS data.

Prior to the pandemic, WHO and UNICEF created a guidance module on RMNCH for program managers to increase country capacity to collect, analyze, and visualize data from RHIS [10]. The module was disseminated through a series of regional workshops through a train the trainer approach. Further plans to promote and refine the module based on country user feedback and to link the module with a companion module on community health data were interrupted by the pandemic. This module is part of a suite of WHO guidance modules on analysis and use of health facility data that cover a range of topic areas, and are complementary to the WHO harmonized health facility assessment tools and the SCORE package that enables countries to track progress with health information system strengthening [11]. The COVID19 specific RMNCH module described above has been added to this compendium of modules to provide countries with guidance on a prioritized set of RMNCH indicators to monitor during emergency and non-emergency times. Numerous other initiatives including the Countdown to 2030 regional workshops and efforts supported by the Global Financing Facility were launched pre-pandemic to foster country data ownership and strengthen local capacity in LMICs to produce and use RHIS data. This BMC Health Services Research series of papers helps further advance this capacity building agenda and provides evidence on ways to improve the quality of RHIS in LMICs. Results from the studies on how best to conduct data quality assessments and to calculate coverage estimates using RHIS data, for example, will be incorporated into the WHO-UNICEF RMNCH guidance module.

These joint efforts, while a promising start, are not enough, especially in fulfilling the principle of country leadership and ownership around data and accountability. UN agencies and other development partners often inadvertently cause fragmentation in data systems at the country level when their investments in RHIS and in capacity building create parallel systems or are duplicative. To help address this problem, the Every Woman Every Child Global Strategy for Women’s, Children’s and Adolescents’ Health [12] is being integrated into the Global Action Plan for Healthy Lives and Well-being for All. This Global Action Plan brings together 13 multilateral health, development, and humanitarian agencies to align efforts, leverage resources, and better support countries to accelerate progress towards the health-related Sustainable Development Goals (SDGs) [13]. Another avenue for fostering UN agency and development partner alignment on health information system strengthening at global, regional, and country levels is the Health Data Collaborative launched after the 2015 Measurement Summit [14]. The Health Data Collaborative provides a platform for coordinating partner activities to best support country owned plans for collecting, storing, analysing, and using health data, and brings together existing efforts including the Health Information Systems Programme supported DHIS2 project and the RHIS Network [14]. A new working group has been formed under the Health Data Collaborative umbrella to improve RHIS in countries. The Health Data Collaborative is also working closely with the Global Action Plan accelerator group on data and digital health, particularly in four priority countries to ensure partner activities support country plans to improve their health information systems including adopting electronic registers and other digital innovations (Malawi, Kenya, Nepal, Uganda).

These efforts are a step in the right direction, but more work to align donor investments and to avoid the creation of parallel reporting systems for single diseases or issues is needed, especially in an expected tighter funding environment because of the economic downturn caused by COVID19. Donors have a responsibility to better coordinate and increase their investments in country RHIS. Development partners should ramp up activities with country institutions that build local capacity around data collection, analysis, dissemination, and use. UNICEF and WHO should join forces to develop long overdue standardized registers for RMNCH services as a fundamental step towards strengthening RHIS systems, improving the comparability of RHIS data across settings and time, and reducing the reporting burden on front-line health workers. Perhaps most importantly, LMICs need to allocate more resources to health information system strengthening and to increase the engagement of their own institutions in data analysis and interpretation. Building resilient RHIS systems across LMICs will require governments, donors, and development partners to each play their part and to commit to effective coordination.

The COVID19 pandemic has made it clear that the benefits of resilient RHIS systems far outweigh the costs. Strong RHIS systems will improve the ability of countries to swiftly mount evidence-based strategies to future crises while maintaining essential RMNCH services, and to tailor programs and service delivery strategies to local settings. The pandemic has also illustrated deep global interconnectedness and how health problems in one setting if not addressed can spill across borders. Thus, building strong RHIS systems around the world is an important step to improving health for all.

## Data Availability

Not applicable.

## Abbreviations

RMNCH: Reproductive Maternal Newborn Child Health
RHIS: Routine Health Information System
WHO: World Health Organization
UNICEF: United Nations Children’s Emergency Fund
DHIS2: District health information system 2
SCORE: Survey, Count, Optimize, Review, Enable
LMICs: Low-and middle-income countries
